# First Report of a Severe Outbreak of Aujeszky’s Disease in Cattle in Sicily (Italy)

**DOI:** 10.3390/pathogens9110954

**Published:** 2020-11-17

**Authors:** Flavia Pruiti Ciarello, Maria Teresa Capucchio, Dorotea Ippolito, Elena Colombino, Lucia Rita Maria Gibelli, Michele Fiasconaro, Ana Maria Moreno Martin, Vincenzo Di Marco Lo Presti

**Affiliations:** 1 Istituto Zooprofilattico Sperimentale “A.Mirri” della Sicilia, Via G. Marinuzzi 3, 90129 Palermo, Italy; pruitiflavia@outlook.it (F.P.C.); michele.fiasconaro@izssicilia.it (M.F.); vincenzo.dimarco@izssicilia.it (V.D.M.L.P.); 2 Department of Veterinary Sciences, University of Turin, Largo P. Braccini 2, 10095 Torino, Italy; mariateresa.capucchio@unito.it (M.T.C.); elena.colombino@unito.it (E.C.); 3 Istituto Zooprofilattico Sperimentale della Lombardia e dell’Emilia Romagna “B.Umbertini”, Via Bianchi 9, 25124 Brescia, Italy; luciarita.gibelli@izsler.it; 4 National reference Centre for Aujeszky Disease-Istituto Zooprofilattico Sperimentale della Lombardia e dell’ Emilia Romagna “B.Umbertini”, Via Bianchi 9, 25124 Brescia, Italy; anamaria.morenomartin@izsler.it

**Keywords:** cattle, outbreak, Aujeszky’s disease, pseudorabies, Suid Herpesvirus 1, immunohistochemistry

## Abstract

Aujeszky’s disease in cattle is caused by Suid herpes virus 1. The natural infection has been reported worldwide in bovine species and it is related to direct and indirect contact with infected pigs, which represent the main reservoir of the virus. Here, it is reported the first documented outbreak of Aujeszky’s disease in cattle in Sicily (Italy). Severe itching and nonspecific neurological symptoms were the main reported clinical signs. No characteristic gross and histological features were reported other than cutaneous lesions caused by excessive pruritus and hyperaemia, haemorrhages and inflammation in the central nervous system. Diagnosis was confirmed by real time PCR and immunohistochemistry on the nervous tissue. The route of infection remained unknown, but serological data observed in pigs living in close cohabitation with cattle revealed a circulation of a wild strain of the virus in the area. This study contributes to a better knowledge of this disease in a non-conventional host and suggests the need to increase the prophylaxis control plans in specific breeding contexts.

## 1. Introduction

Aujeszky’s disease (AD) is a notifiable disease caused by Suid Herpes Virus 1 (SuHV-1) also known as Pseudorabies virus (PRV) that is a member of the Herpesviridae family, genus Varicellovirus [1]. Pigs (*Sus scrofa domesticus*) are considered the natural hosts, serving as a source of the virus to other animal species, including ruminants, carnivores and rodents. In Europe, the wild boar is considered the main reservoir of AD which is endemic in domestic free-range pigs [2,3,4,5,6]. There is still no consensus on the zoonotic potential of AD, although positive cases have been documented especially in people working in close contact with pets and farm animals (e.g., laboratory technicians, veterinarians, cattle and pig farms employees) [7,8,9,10,11,12,13,14]. Many cases of AD have been reported in cattle throughout the world [8,15,16,17,18,19,20,21,22,23], and although they differ in terms of clinical manifestation (presence and site of itching), an epidemiological relation to direct or indirect contact with pigs infected with a wild-type of SuHV-1 seems to be constant [20]. AD in cattle is sporadic but lethal as the recovery is extremely rare [17,20,24,25]. Infection occurs generally by direct contact, via faecal-oral route or by aerosol, but given the high stability of the virus in the environment, indirect infection by exposure to infected fomites is also described [26]. Additionally, accidental exposure to the modified live vaccines developed for swine (e.g., through contaminated syringes) may cause disease in ruminants [27,28]. The clinical symptoms are due to the neurotropic nature of the SuHV-1, which after a first replication phase in peripheral neurons, spreads centripetally to the central nervous system (CNS). The incubation period in cattle varies from 3 to 6 days. The animals might die suddenly without premonitory signs or develop local pruritus as a main clinical sign and/or aspecific symptoms (high body temperature, discomfort, continuous bellowing, whirling around, convulsions, opisthotonos), followed in any case by death within few days [21,27,28,29]. AD diagnosis in cattle is often based on clinical signs and possible contacts with pigs reported in anamnesis. Although serology serves as a useful screening tool in pigs, detection of antibodies directed to SuHV-1 is not commonly used in cattle as well as in other non-natural hosts, as most of the animals die before detectable serum antibodies are produced [26,30]. The aim of this study is to describe the clinical, diagnostic and pathological aspects of the first reported AD outbreak in cattle in Sicily (Italy).

## 2. Results

### 2.1. Description of the Outbreak

**Figure 1:**
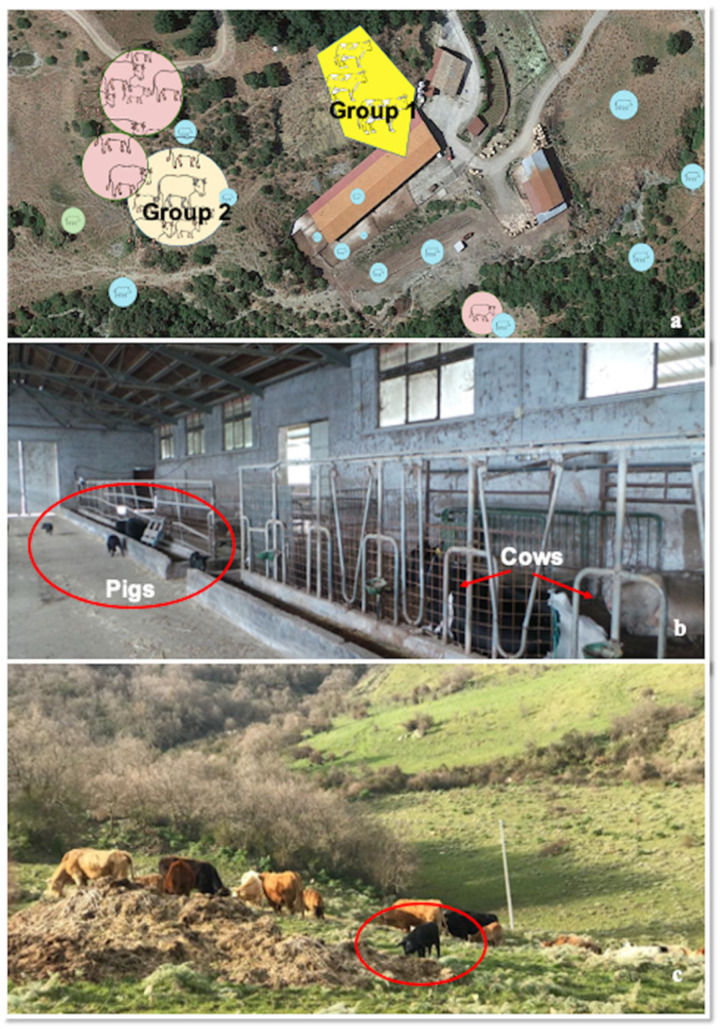
Farm structure. (**a**) animal distribution in the farm (pig-cattle breeding); Group 1: cattle kept in the barn; Group 2 cattle at pasture; the distribution of pigs (icons circled in blue) is identified both in the stable and in the pasture.; (**b**) close cohabitation between pigs (circled) and cattle (arrows) (group 1) in the barns and contamination of feeders by pigs; (**c**) pasture shared between pigs (circled in red) and cattle (group2).

In March 2019, the operational unit of Istituto Zooprofilattico Sperimentale of Sicily (Area Barcellona P.G.) performed four inspections in a mixed farm of cattle and pigs in the town of Cesarò (Lat. 37.863056 Long.14.658611) in the Nebrodi Park area (Sicily, Italy) as an AD outbreak in cattle was strongly suspected. The farmer reported sudden death of two cows two-tree days after the onset of intense itching. The farm consisted in an extensive farming system with a mixed bovine-porcine herd in which a total of 58 cattle (45 cows, 12 calves and 1 bull) shared pasture, feeding and resting areas with around 300 Nebrodi Black pigs. At the time of the outbreak, pigs were partly kept in the barn and partly distributed in the grazing areas, in close cohabitation with cattle (Figure 1, Panels a–c). Immunoprophylaxis for AD in pigs was historically performed in the farm using an attenuated live vaccine (Ad live Suivax—Fatro) and the last administration was recorded 20 days before the cattle outbreak. Despite the farm was not officially AD free, no related symptoms were recorded in pigs. Cattle herd consisted in two separate units: the first (Group 1) composed by 1 bull and 11 lactating cows, kept indoor in the stable and the second (Group 2) composed by 34 cows and 12 calves, kept in a grazing area adjacent to the stable and fed mainly on pasture (Figure 1, Panel a). The water supply was guaranteed by a unique source originating from a lake and conveyed inside the stable for Group 1, while Group 2 was watered by collection tanks filled daily by the breeder. No biosecurity measures were adopted in the farm in order to avoid contact between cattle and pigs or to prevent contacts with wildlife. At the time of inspection, clinical symptoms developed almost simultaneously in four non-pregnant lactating cows (60–70 days post-partum) belonging to Group 1. Pruritus was the predominant sign followed by death within 2–3 days. Corticosteroid and parasiticide were administered to contain the itch but no recovery was achieved. The death occurred spontaneously during the night in 3 out of 4 cows, whereas the remaining one was slaughtered. The owner, as a preventive measure, decide to slaughter the remaining 7 lactating cows of Group 1, but no post-mortem examination (PME) was carried out. In Group 2, clinical symptoms developed simultaneously in 2 cows, that both died after a few days. Despite no symptoms were recorded, the remaining 32 cows of Group 2 were slaughtered, except for 12 young calves and one bull. At the slaughterhouse, the ante-mortem inspection detected neurological symptoms in three cows. In total spontaneous death occurred in seven cows within 9 days.

### 2.2. Clinical Examination

**Figure 2:**
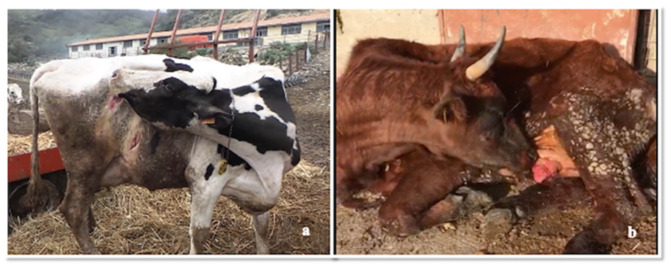
Clinical manifestation of severe itching: (**a**) flank region (**b**) breast.

**Figure 3:**
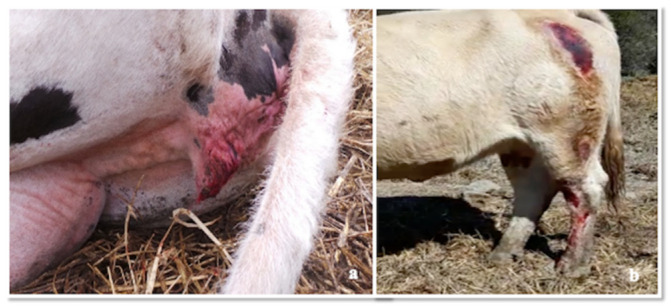
Posterior regions of the body where self-mutilation-induced trauma has occurred due to intense itching: (**a**) vulva (**b**) flank region and distal limbs.

At general clinical examination, cattle showed aspecific symptoms such as anorexia, depression, restlessness, dehydration (15%), hyperthermia (39–40 °C), increased heart and respiratory rate with opaque and hyperaemic ocular, buccal and vaginal mucosa. Moreover, severe itching was the most characteristic and constant symptom with hyperexcitability and continuous licking/scratching of the distal parts of the hind limbs, flank and udder (Figure 2, Panels a,b; Video S1). At this level, severe lesions with haemorrhages, haemorrhagic suffusions and crusts were found, being worse in the nipples. Open wounds, cutaneous excoriations and oedema due to self-trauma, were also evident in perineal area and vulva (Figure 3, Panel a,b). Between an itch attack and the other, the animals were exhausted, with the head bent sideways in the position of self-heard. Unspecific neurological symptoms were also present, such as stiff gait, hind limb hypometria and proprioceptive deficit. Progressively the animals showed dyspnoea, hypersalivation, spasms of abdominal muscles, foaming and tympanicity; subsequently, these symptoms worsened, with teeth grinding and kyphotic posture as a result of evident abdominal pain due to colic-like syndrome with flatulence and complete impairment of digestive functions. In the final stages, the presence of an abundant salivary drain (no sialorrhea) was probably associated with the decrease of the swallowing reflex. Finally, the animals persist in recumbency and died within 2 or 3 days from the onset of severe symptoms. Besides, other potential causative agents of itching (mange and pediculosis) were excluded through the inspection of skin/skin lesions and by performing a skin scraping.

### 2.3. Serological Data

Data from cattle serum samples are reported in Table 1; antibodies targeting the gB were detected using both in-house and a commercial ELISA kit (positivity detected in 89.8% and 83.3% of the cases, respectively). Nevertheless, all samples resulted negative for anti-SuHV-1 gE antibodies. At Virus neutralization test (VNT), only two samples showed positivity with neutralization titres of 16/32 and 64/128 in the two replicates. The serological results of samples taken from pigs (Table 2) were positive for antibodies against both SuHV-1 gB and gE, confirming the circulation of a wild-type strain in the pig farm. The results of the serum biochemical tests, however, did not present any noteworthy alteration.

### 2.4. Post-Mortem Investigations

#### 2.4.1. Necropsy

**Figure 4:**
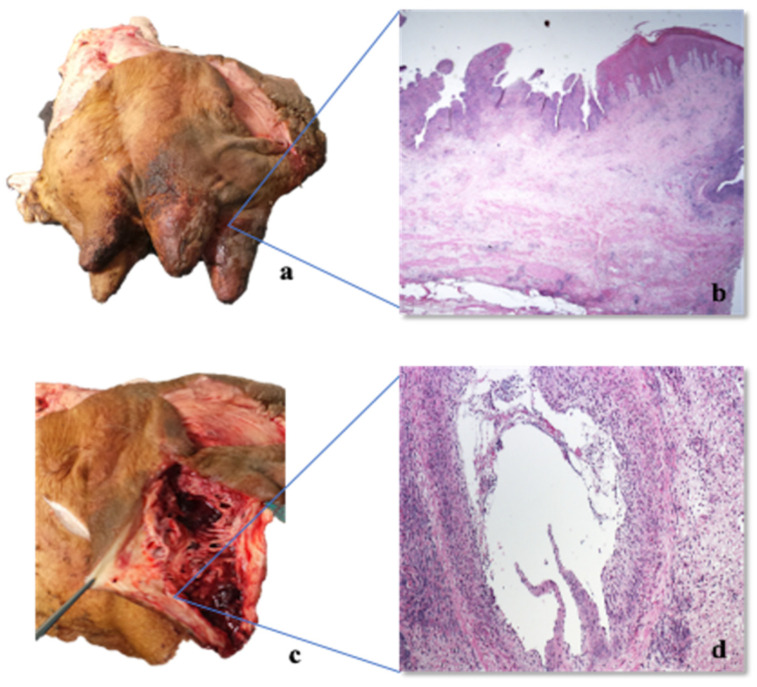
Udder, auto-mutilation due to intense itching: (**a**,**c**) gross traumatic lesions, such as crusts, haemorrhagic suffusions and severe traumatic haemorrhage in the nipple; (**b**) Udder (itching area) HE 20×, severe necrosis of the epithelium; (**d**) Udder, HE 100× dermis, severe pyoderma and severe and diffuse necrotizing vasculitis.

**Figure 5:**
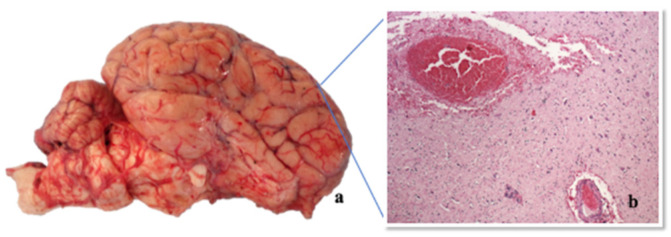
Telencephalon: (**a**) Gross macroscopic evidence of hyperaemia of leptomeninges; (**b**) Telencephalon, HE 100×, serious scattered haemorrhages in the grey and white substance, minimal multifocal gliosis, minimal non-purulent perivascular sleeves.

**Figure 6:**
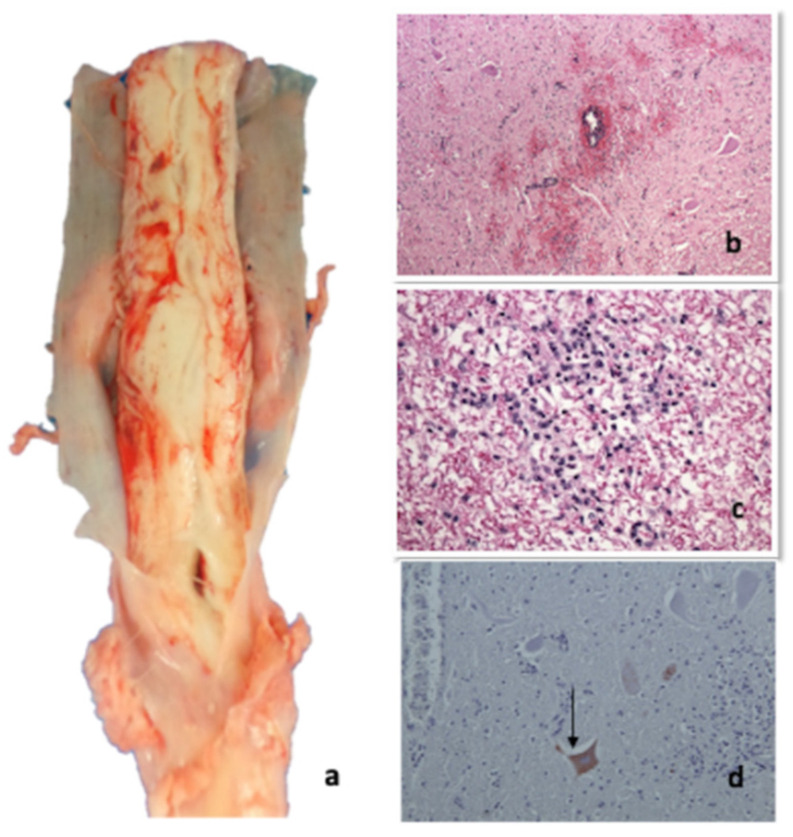
Cord: (**a**) gross macroscopic evidence of hyperaemia of the spinal cord; (**b**) spinal cord HE 100×, serious scattered haemorrhages in the grey and white substance, minimal multifocal gliosis, minimal non-purulent perivascular sleeves; (**c**) spinal cord, HE 400×, gliosis in the white matter; (**d**) spinal cord, HE 200×, Immunohistochemistry with pool of three MAbs (clone 1F2, 2E12 and 3D5) multifocal granular SuHv-1 positivity in the cytoplasm of the neurons (arrow).

All the cows showed multiple skin abrasions/laceration and oedema in the itchy areas, mainly in the hindquarter (tights and distal regions of the hindlimb), mammary gland, nipples (Figure 4, Panels a,c), vulva and perineum. Meningeal hyperaemia involving telencephalon, cerebellum (Figure 5, Panel a), lumbar and thoracic spinal cord were also detected (Figure 6, Panel a). Sero-haemorrhagic fluid was detected in the abdomen, thoracic cavity and pericardium along with epicardial petechial, multifocal haemorrhages in spleen and kidney, splenomegaly and hepatomegaly.

#### 2.4.2. Histopathological Investigations

The organs of the two examined animals (Group 1—Cows 1–2) showed similar histopathological lesions, even if one case (Cow-2) was more severe than the other was. In order to avoid repetition, the two cases were described together. At histological examination, cerebral cortex, pons and rostral brainstem showed mild to moderate multifocal haemorrhages with perivascular oedema without inflammatory response both in white and grey matter. Mild multifocal haemorrhages were present in the meninges (Figure 5, Panel b). Both in thoracic and lumbosacral spinal cord mild to severe multifocal haemorrhages, mild, multifocal non-suppurative perivascular cuffings composed of lymphocytes, macrophages, and a few plasma cells, and diffuse gliosis were observed mainly in the grey matter (Figure 6, Panels b–d). Tonsils, retropharyngeal and supramammary lymph nodes showed mild reactive hyperplasia associated with rarefaction of the lymphatic centres and focal macrophages proliferation. Besides, retropharyngeal lymph nodes presented moderate multifocal necrotizing vasculitis with hyperaemia, haemorrhages and multiple calcified pyogranulomas. Mammary glands were characterized by mild multifocal suppurative alveolitis and haemorrhages. The skin of the udder, one of the itchiest regions in these animals, showed severe epithelial ulceration, haemorrhages and diffuse acute suppurative inflammation. In the dermis, severe diffuse necrotizing vasculitis surrounded by polymorphonuclear cells was also observed (Figure 4, Panels b,d). Mild non-suppurative epicarditis and myocarditis with moderate multifocal haemorrhages and necrotizing vasculitis were detected in the heart. Mild multifocal perivascular non-suppurative hepatitis with moderate multifocal vacuolar degeneration of the hepatocytes and scattered haemorrhages were observed in the liver. Spleen presented severe and diffuse white pulp rarefaction with haemolysis, hemosiderosis and local macrophages proliferation while kidney showed mild multifocal non-suppurative perivasculitis associated with renal medullary haemorrhages and pigmentation into the tubular epithelium. Caudal brainstem, hippocampus, cerebellum and lungs did not show any histopathological alterations. Immunohistochemistry revealed multifocal granular SuHV-1 positivity in the neuronal cytoplasm of the spinal cord of Cow 1 (Table 1) using both antibodies. The clones (1F2 and 3D5) used individually on the animal’s spinal cord revealed equal reactivity in the cytoplasm of the neurons. No histopathological lesions/immunohistochemical positivities were detected in the brain of 12/32 cows belonging to group 2.

### 2.5. Microbiological and Molecular Data

The real-time PCR assays for gE and gB SuHV-1, resulted positive only on a sample of spinal cord (Thoracic tract) of one symptomatic cow (Cow 1), while resulted negative on all nasal swabs of pigs and all nasal swabs and EDTA blood samples collected from cows (Table 1). However, the real-time PCR positive sample resulted negative for virus isolation in PK15 cells.

**Figure 7:**
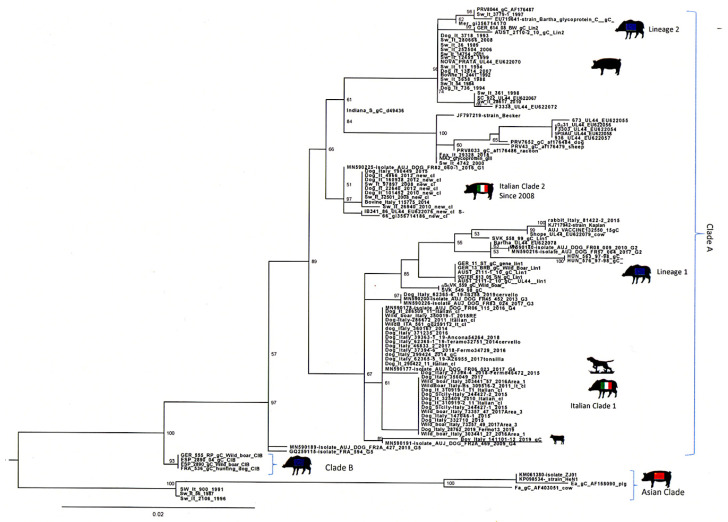
Phylogenetic tree based on partial sequencing of the UL44 gene. The tree was obtained using the maximum likelihood method and the HKY85 + I + G model with 1000 bootstrap replicates. The bootstrap percentage values are indicated at nodes. The Italian bovine sequenced are underlined.

Phylogenetic analyses of the Italian sequence based on the partial sequencing of the UL44 and US8 genes encoding the gC and gE protein, respectively, were performed by comparison with other field and reference SuHV-1 sequences. Blast analysis showed the highest identity rates (98.94%) with Italian and French sequences. The first sequences belonged to the Italian clade 1 [4], which grouped together sequences from wild boars and hunting dogs. The second ones came from French dogs originating from the south-eastern part of the country near the Italian border [31]. The percentage of identity towards SuHV-1 strains currently circulating in Italian domestic pigs and farm dogs (Italian clade 2) was instead lower (98%). The phylogenetic tree of the gC gene showed the presence of three clades: A, B and Asian (Figure 7). The Italian strains all belong to the clade A with the exception of three strains isolated in the 1990s that belong to the Asian clade. Within this clade, the Italian sequences were distributed into two groups based on their origin. The sequences originating from wild boar and hunting dogs formed the Italian clade 1 closely related to the French dogs of group 4. Those originating from pigs and farm dogs instead formed the Italian clade 2. The Italian bovine sequence clustered with a sequence of a 2009 French dog and was closely related to the Italian clade 1 (Figure 7) [31]. The US8 gene encoding the gE protein was found to be a very conserved gene and therefore much less informative than the UL44 gene. The low number of information sites has therefore led to a phylogenetic tree with not very high bootstrap values.

**Figure 8:**
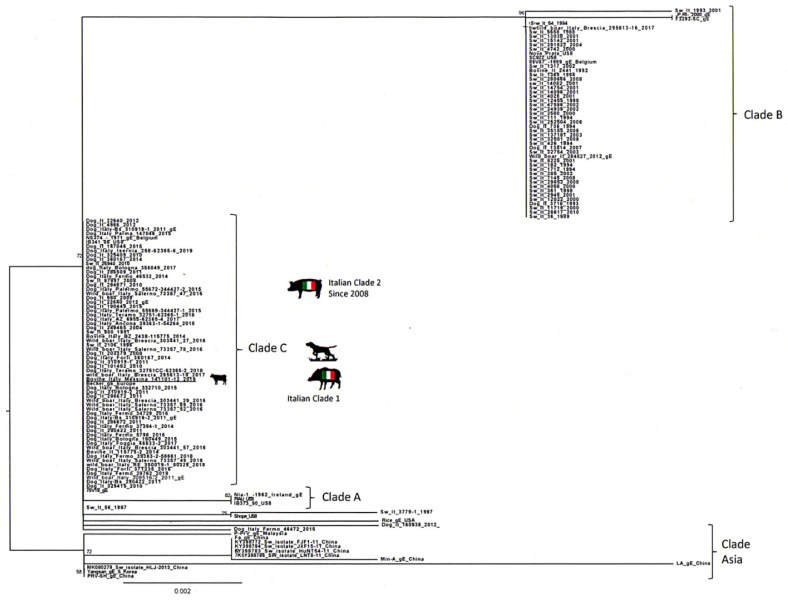
Phylogenetic tree based on partial sequencing of the US8 gene. The tree was obtained using the maximum likelihood method and the HKY85 + I + G model with 1000 bootstrap replicates. The bootstrap percentage values are indicated at nodes. The Italian bovine sequenced are underlined.

Phylogenetic analysis of the US8 gene (Figure 8) revealed the presence of 4 clades, named A, B, C and Asia, as reported in the study of Fonseca et al. [31]. Our bovine sequence was placed in the clade C together with all the Italian sequences that in the UL44 phylogenetic tree formed the Italian clades 1 and 2. Interestingly, this clade was reported by Fonseca et al. [32] as a new clade that included only one strain isolated in Brazil in 1986.

### 2.6. Retrospective Analysis on the Prevalence of Aujeszky Disease in Pigs in Sicily

**Figure 9:**
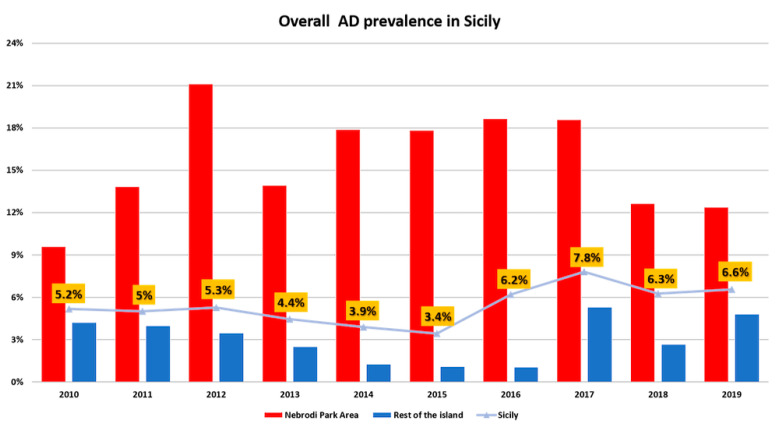
The overall prevalence of Aujeszky’s disease in pigs in Sicily. To note the constant higher positivity rate registered in the Nebrodi park area when compared to the rest of the island and the average prevalence recorded in Sicily during the years to 2010 to 2019.

In the years considered, the average prevalence of AD in Sicily was 5.41% (CI 95% 4.58–6.22%). The trend registered each year is illustrated in the Figure 9, showing a decrease from 2012 to 2015 (with a minimum value of 3.44%, CI 95% 2.43–4.46% in 2015), and a sharp increase the following year, peaking in 2017 (7.81% CI 95% 6.54–9.08%). However, the prevalence levels referred to the last year examined (2019) decreased back to the percentages registered in 2010 (prevalence of 5.41%, CI 95% 5.51–7.60%). We consequently considered the average prevalence of AD per province, during the period 2010–2019. The disease was absent in the province of Caltanissetta during the period considered, and present in low levels (below 2%) in the provinces of Agrigento, Palermo, Ragusa, Siracusa and Trapani. The provinces with the higher prevalence rates were Enna, Catania and Messina (3.81%, 9.57% and 12.20% respectively). Considering the prevalence of AD in the Nebrodi Park Area and in the rest of the island, the overall positivity in Nebrodi Area is significantly higher (χ^2^ 403,025 with *p* = 0) compared to the remaining territory (ORs 5.50 CI 95% 4.58–6.60).

## 3. Discussion

The present study reports a severe outbreak of AD in cattle, in which the first suspicion was based on typical itching, neurological symptoms and evidence of direct contact with pigs. The confirmation of AD was made based on the real-time PCR and immunohistochemical results. The symptoms described in the present study were similar to those reported in the literature in cattle and were mainly characterized by neuropathic itch, considered a typical sign of AD in non-natural hosts [8,17,20,21,22,33]. Although the presence of itching may facilitate the diagnosis of AD, a differential diagnosis is required for psoroptic and sarcoptic mange and pediculosis. In this case, the skin scraping results as well as the clinical examination excluded all the differentials diagnosis mentioned above. Experimental infection studies with SuHV-1 in cattle have shown that the extent of viral spread in the host body and the resulting clinical picture is determined by the virus penetration site [34]. Ruminants can be infected after intradermal, subcutaneous, intranasal, oral route or by introduction through the vaginal mucosa [35]. In case of virus exposure through the oral, rectal and vaginal mucosa, pruritus typically develops in the head, shoulder, flank, hind quarters and perineum [34], whereas, head and neck are mainly involved when infection occurs trough the nasal mucosa or respiratory tract [34]. The differential location of pruritus and pathological findings suggest different distribution of the virus [33]. The pruritus indeed usually develops at the point of inoculation of the virus, reaching the related segment of the spinal cord from the peripheric nerve termination. In the present case, the presence of the virus was detected in the thoracic tract of the spinal cord, and the main lesions were recorded in the caudal portion of the body (hind limbs, udder, perineal and vulvar region) but the route of infection remains still unknown. The heavy contamination of feeders, grazing and watering areas, with swine faeces and urine as well as the direct and continuous contact between the two species, raises the hypothesis that the transmission took place through skin abrasions or vaginal and oral mucosa by direct contact with excretion and secretion from infected pigs.

AD diagnosis was supported by the serological finding of a wild type SuHV-1 strains circulating in vaccinated pigs, demonstrating also that the vaccination plan was unsuccessful in the farm of the present study. Furthermore, considering the absence of barriers preventing contacts with wildlife and the high similarity between the viral strain detected in cow and the viral strains previously identified in wild boars, it is not possible to rule out a direct transmission from wild boars/feral pigs to cattle (Figure 7 and Figure 8). Thus, the area where the farm is located shows a high density of wild boars and feral pigs’ population, which have been widely recognized as AD reservoirs all over Europe [2,3,4,5,6]. Furthermore, the highest similarity between SuHV-1 strains isolated in hunting dogs (unpublished data- Istituto Zooprofilattico Sperimentale of Sicily) in the same territory and the strain isolated in the current study (Figure 7) suggests that there could be multiple exposure sources along with interspecies virus transmission. In literature, there is no evidence of characteristic gross lesions rather than cutaneous unravelling lesions caused by excessive pruritus and following licking, and hyperaemia of the central nervous system [20,36]. The present case confirms the previous reports, as the main findings were hyperaemia of the leptomeninges, especially in the telencephalon and spinal cord, associated with secondary lesions of the skin [23]. Histopathological alterations mainly involved the cortex, brainstem and spinal cord with haemorrhages, non-suppurative perivascular cuffings and gliosis particularly affecting the grey matter, as already reported in the literature. These findings reflect the neurotropic and epitheliotropic nature of the virus [37]. The suppurative lesions and the necrotizing vasculitis recorded in the skin of the itchy regions are due to self-injuries and secondary to acquired bacterial infections. In Italy, the current national control program of AD in pigs is based on serological surveillance, sanitary prophylaxis and vaccination (GURI—Decree 01/04/1997 Art.1). Moreover, the use gE deleted live attenuated vaccines for the breeding pig category was allowed experimentally for two years (DDMM 30/12/2010 and 4/10/2011) and then authorized in 2013 for breeding pigs but with maximum biosecurity condition (Ministry of Health, circular of 17/05/2013). Unlike other Italian regions, in Sicily there is no regional control plan, therefore the national plan is applied. However, in the Nebrodi area, where the present outbreak occurred, the majority of pigs are bred in extensive or semi-extensive farming systems with traditional management in which transhumance, mountain grazing as well as sharing of watering and feeding areas are common. In this context the application of adequate bio-safety measures is challenging. The grazing areas of the park are periodically exploited by different breeders, involving a mixture of farms and species (sheep, goats, horses, donkeys, pigs) in direct contact with wildlife (foxes, martens, wild boars, feral pigs, wild cats, etc.). Moreover, retrospective analysis of AD prevalence in pigs in Sicily, showed that between 2010 and 2019, the AD prevalence constantly increased (Figure 9) in the Nebrodi park, showing a significantly higher prevalence compared to the rest of Sicily and suggesting that the disease control and surveillance plan it is not effective. To state, the severe outbreak presented in this report makes an important contribution to the knowledge of clinical, pathological and diagnostic features of AD in cattle. Moreover, given the possibility of disease transmission and diffusion between different species, outbreaks of AD in other non-natural hosts might represent a sentinel for the persistence of the disease in multi-host breeding contexts.

## 4. Materials and Methods

### 4.1. Anamnesis, Clinical Examination and Sampling

During the inspections carried out on the farm, all the anamnestic and epidemiological information were recorded and evaluated in the diagnostic process. Complete clinical examination was performed on all the symptomatic subjects. Evaluation of the basic clinical parameters (temperature, hydration status, heart and respiratory rate), an inspection of skin and skin appendages and evaluation of neurological parameters were carried out. Concerning the non-symptomatic cattle and the pigs, remote clinical examination and recording of the anamnestic information from the breeder were pursued. Blood samples (serum and whole blood) from 39 cows (symptomatic and slaughtered cows) were collected and sent to the C.R.M.A. (National Reference Centre for Aujeszky’s disease) in order to perform serological and virologic investigations. An aliquot of serum was sent to the IZS laboratories for biochemical investigations. Moreover, serum samples from 16 fattening pigs and 32 breeding pigs were also collected. The nasal swabs were collected in Minimum Essential Medium (MEM) respectively, from 39 pigs (fattening and breeding pigs) and 41 cows (symptomatic and asymptomatic cows), for biomolecular investigations. At the same time, a retrospective analysis of the seroprevalence of SuHV-1 in Sicily was carried out to provide epidemiological data on the disease.

### 4.2. Serological Analysis

Serum samples collected from cattle and pigs were tested for detection of antibodies against gB and gE antigens of SuHV-1. The presence of anti-gE antibodies was determined using a commercial ELISA kit (IDEXX^®^ PrV/ADV gE Ab test, IDEXX, Hoofddorp, The Netherlands) according to the manufacturer’s instructions. The presence of gB antibodies was investigated using two different assays, a commercial ELISA kit (IDEXX^®^ PrV/ADV gB Ab Test, IDEXX, Hoofddorp, The Netherlands) and an in-house blocking ELISA kit performed as previously described by Cano-Manuel et al. [38]. Since ELISA tests are set up to test sera from pigs and are not validated for bovine sera, all bovine samples were also tested using a virus neutralization test (VNT) against SuHV-1 (NIA-3 strain). The VNT was performed in 96-well cell culture plates, and sera were tested in duplicate in four final dilutions from 1:2 to 1:16. The plates were further incubated at 37 °C for 4 days. Each set of plates included virus control (target titre 100 TCID50/50 µL) and cell control. The neutralizing titer of a serum was expressed as the highest initial dilution that brings complete neutralization of the cytopathic effect (CPE) of the virus in 50% of the wells. Three control sera were included in both the gB ELISA and the VNT. High positive (national reference standard serum 7183) and negative (national reference standard serum 571) control sera were used. A low positive control (national reference standard serum 1355), previously calibrated against the international reference standard serum (ADV1) was also included. Both national and international reference sera were scored positive at a dilution of 1:2, as stated in ANNEX III of Standards for Aujeszky’s disease serological tests (DIR 2008/185/EC).

### 4.3. Post-Mortem Investigations

#### 4.3.1. Necropsy

Three dairy Friesian adult cows (3–5 years) belonging to Group 1 (Cow 1-2-3) and one mixed breed dairy adult (5 years) cow belonging to Group 2 (Cow 7) were submitted to anatomic pathological investigations. Unfortunately, only two carcasses (Cows 1–2) were available for complete necropsy immediately after death, while on the other two cows (Cows 4–8), only a partial necropsy was performed, limited to external examination of the itching sites and evaluation of the nervous system. During necropsy, samples of the skin of the itchy regions (udder skin), tonsils, retro-pharyngeal lymph nodes, lung, heart, liver, spleen, kidney, mammary glands, supramammary lymph nodes, brain (pons, brainstem, hippocampus, cerebellum), thoracic and lumbosacral spinal cord were collected from 2 cows (Cows 1, 2) of the four examined cows for further investigations. An aliquot of all these samples was fixed in 10% buffered formalin and sent to the Department of Veterinary Sciences, University of Turin (Italy) for histopathological investigations. Another aliquot of the same samples was used to perform aerobic and anaerobic bacterial cultures and a third aliquot was frozen at −80°C and sent at C.R.M.A. for virologic investigations. Moreover, the brains of 12/32 cows belonging to Group 2 were collected at the slaughterhouse and submitted to histopathological and immunohistochemical investigations.

#### 4.3.2. Histopathological and Immunohistochemical Investigations

All the tissue samples were routinely embedded in paraffin wax blocks, sectioned at 5 μm thickness, mounted on glass slides and stained with Haematoxylin and Eosin (HE) according to standard protocols. Histopathological changes were evaluated by light microscopy. Selected sections of two animals (Cows 1–2) (297/19 B; 297/19 C) were sent to the Istituto Zooprofilattico Sperimentale of Lombardia and Emilia Romagna to perform immunohistochemistry. A pool of three MAbs (clone 1F2, 2E12 and 3D5) diluted 1:800 was used: the MAb 1F2 recognized the gC protein, whereas the other two (2E12, 3D5) recognized the gE protein [39]. In addition, clone 1F2 and 3D5 were individually tested on animal’s spinal cord (297/19 B). Immunohistochemical reaction was visualized through a secondary detection system Novolink (Novocastra).

### 4.4. Molecular and Virologic Investigations

Genomic DNA extraction from nasal swabs (cows and pigs), whole blood and all tissue samples collected during necropsies (Cow 1–2) was performed using a RNeasy kit (Qiagen, Hilden, Germany) and therefore the presence of the SuHV-1 DNA was determined by a real-time PCR based on the specific detection of the gB gene as described by Yoon et al. [40]. The presence of the gE gene was also performed on positive gB PCR samples to differentiate between wild-type and vaccine SuHV-1 strains.

The UL44 gene encoding the glycoprotein gC and the US8 gene encoding the glycoprotein gE were partially sequenced using the PCR protocols described by Fonseca et al. [32]. The PCR products were purified using a QIA quick Gel Extraction Kit (Qiagen, Inc., Valencia; CA, USA). DNA sequencing was performed using a Big-Dye Terminator Cycle sequencing kit (Applied Biosystems, Foster City, CA, USA) with the same primers used for amplification. The sequencing reactions were run by capillary electrophoresis on an automatic sequencer (ABI 3130 Genetic Analyser; Applied Biosystems^®^, Foster City, CA, USA). The sequences were edited using the SeqMan program (DNASTAR, Madison, WI, USA). For phylogenetic analyses, SuHV-1 sequences were compared to reference sequences and wild-type SuHV-1 strains available in GenBank. The phylogenetic tree was constructed using the maximum likelihood (ML) method within the IQ-tree software [41] with bootstrap analyses involving 1000 replicates. The sequence alignment was performed using the ClustalW method (DNASTAR, Madison, WI, USA) and was manually optimized. The best-fit model of the nucleotide substitution was determined using the jModelTest v.0.1.1 [42]. All the models were compared using two criteria: the Akaike’s information criterion (AIC) and the Bayesian information criterion (BIC). The preferred model was the HKY85+I+G model. The topologies were verified with the neighbour-joining method and the Kimura two-parameter model using MEGA 6 [43]. Positive PCR samples were inoculated into porcine kidney cell line PK-15 for virus isolation. The presence of the SuHV-1 antigen was detected in the infected cell line using immunoperoxidase with two MAbs specific to gB and gE proteins, respectively [39].

### 4.5. Retrospective Analysis on the Prevalence of Aujeszky Disease in Pigs in Sicily

Seroprevalence data of AD in Sicily in the period between 2010 and 2019 were retrospectively examined. The samples came from an average of 1001 farms per year scattered in the nine provinces of Sicily (Agrigento, Caltanissetta, Catania, Enna, Messina, Palermo, Ragusa, Siracusa and Trapani). The samples have been collected according to the legislation in force (Decree 01/04/1997 and further modifications) considering the herd size and the production category. The control program is based on the detection of antibodies against glycoprotein E (gE) using a commercial ELISA kit (IDEXX^®^ PrV/ADV gE Ab test, IDEXX, Hoofddorp, The Netherlands) and performed at the Istituto Zooprofilattico Sperimentale della Sicilia. Data were excluded from the analysis if the sample was reported as “unsuitable for analysis” or “not conclusive”. Firstly, the overall prevalence in Sicily and the different provinces were calculated in the considered period. Thereafter, the prevalence was calculated separately for the Nebrodi Park area and the rest of Sicily. The Park area comprises 24 municipalities scattered in three provinces (19 in Messina, 3 in Catania and 2 in Enna), which provided for a total of 16.868 samples in ten years. The remaining municipalities of Sicily provided 80.143 sera, for a total of 97.011 suitable samples for data collection. The prevalence data from the two areas were analysed by chi-square test (χ^2^) with significant results with *p* < 0.05. Odds-ratios and confidence intervals of 95% were calculated on the average exposure to AD in the two areas in the ten years considered.

### 4.6. Ethic Statement

All sampling procedures performed on live animals comply with good clinical practice and animal welfare. No ethical approval was required for this study, as no experimental procedures were pursued.

## 5. Conclusions

Despite AD has been reported in many countries this is currently the first description of the disease in cattle in Sicily, confirmed by immunohistochemistry and PCR on spinal cord tissue. Phylogenetic analysis revealed that the virus shows the 98.94% of identity with the clade originating from wild boars and hunting dogs. Moreover, pigs living in close cohabitation with cattle showed the presence of antibodies against SuHV-1 gE indicating the circulation of a wild strain in the area.

The cases reported in this study and the serological data suggest that in the Nebrodi Park Area AD is dramatically widespread representing a risk for the entire livestock system; therefore, further control measures should be defined for the particular context described. Further studies should be performed on wildlife and domestic pigs of Nebrodi park area, as well as on cattle herds for better typing the circulating SuHV-1 strains.

## Figures and Tables

**Table 1:** Molecular, serological and immunohistochemistry results of tested cattle.

		Bovine	Nasal Swabs	Blood	Sera				Necroscopy	IHC
Id.	Group	Clinical	SHV-1 RT-PRC	SHV-1 RT-PRC	gB AB ELISA	IDEXX gB AB ELISA	IDEXX gE AB ELISA	VNT SHV-1		
**1**	**1**	**S**	**N**	**N**	**256**	**P**	**N**	**N**	**X**	**P**
**2**	**1**	**S**	**N**	**N**	**64**	**D**	**N**	**N**	**X**	**N**
**3**	**1**	**S**	**N**	**N**	**>256**	**P**	**N**	**N**	**X**	**N**
**4**	**1**	**S**	**N**	**N**	**64**	**D**	**N**	**N**		**N**
5	1		N	N	64	D	N	N		
6	1		N	N	256	D	N	N		
**7**	**2**	**S**	**N**	**N**	**>256**	**P**	**N**	**N**	**X**	**N**
**8**	**2**	**S**	**N**	**N**	**>256**	**NA**	**N**	**N**		
9	2	S	N	N	64	P	N	64/128		N
10	2	S	N	N	16	D	N	N		N
11	2	S	N	N	256	P	N	N		N
12	2		N	N	>256	P	N	N		N
13	2		N	N	64	N	N	N		N
14	2		N	N	256	P	N	N		N
15	2		N	N	64	N	N	N		N
16	2		N	N	16	D	N	N		N
17	2		N	N	>256	P	N	N		N
18	2		N	N	>256	P	N	N		N
19	2		N	N	>256	D	N	N		N
20	2		N	N	16	N	N	N		N
21	2		N	N	>256	P	N	N		
22	2		N	N	256	P	N	N		
23	2		N	N	256	NA	N	N		
24	2		N	N	>256	P	N	N		
25	2		N	N	16	N	N	N		
26	2		N	N	>256	P	N	N		
27	2		N	N	64	P	N	N		
28	2		N	N	>256	P	N	N		
29	2		N	N	64	N	N	N		
30	2		N	N	>256	P	N	N		
31	2		N	N	>256	P	N	16/32		
32	2		N	N	64	P	N	N		
33	2		N	N	>256	NT+	N	N		
34	2		N	N	N	NT	N	N		
35	2		N	N	N	NT	N	N		
36	2		NA *	N	N	NT	N	N		
37	2		N	N	16	NT	N	N		
38	2		N	N	NA	NT	N	N		
39	2		NA	N	16	NT	N	N		

S: symptomatic; * NA: not available +NT: non tested; P: positive; N: negative; D: doubt; the symptomatic cows at the farm are in bold.

**Table 2:** Serological results of tested pigs: detection of antibodies against SHV-1 by ELISA test.

Animal Category	n° Positive /n° of Examined ELISA gE	n° Positive /n° of Examined ELISA gB
Fattening pigs	32/32	32/32
Breeding pigs	12/12	12/12

## Supplementary Materials

The following are available online at https://www.mdpi.com/2076-0817/9/11/954/s1, Video S1: Severe itching of the udder and the nipples in one of the cows affected by AD.
